# Six-session non-operative exercise program yields sustained benefits for up to 18 months in end-stage knee osteoarthritis: a retrospective cohort study

**DOI:** 10.1007/s10067-026-08235-3

**Published:** 2026-06-27

**Authors:** Wing Yip Lee, Linda Man Kuen Li, Naomi Cheuk Ying Chen, Chloe Sze Man Leung, Alan Yin Chung Tsui, Xueyou Zhang, Mingde Cao, Patrick Shu-Hang Yung, Michael Tim-Yun Ong

**Affiliations:** 1 Department of Physiotherapy, Prince of Wales Hospital, Hong Kong SAR, China; 2 Department of Orthopaedics and Traumatology, Chinese University of Hong Kong, Hong Kong SAR, China; 3 CUHK Medical Centre, Department of Orthopaedics & Traumatology, Faculty of Medicine, The Chinese University of Hong Kong, Hong Kong SAR, China

**Keywords:** End-stage knee osteoarthritis, Exercise therapy, Musculoskeletal rehabilitation, Non-operative treatment, Total knee replacement waiting list

## Abstract

**Introduction/Objectives:**

Prolonged waiting times for total knee replacement (TKR) leave patients with end-stage knee osteoarthritis (OA) with persistent pain and functional limitation. We evaluated the effectiveness of a structured physiotherapy-led non-operative program for patients awaiting TKR.

**Method:**

This retrospective cohort included 2,243 end-stage knee OA patients awaiting TKR (October 2021–March 2024). The Structured Non-Operative Treatment Program (SNTP) comprised six sessions delivered over one year, integrating aerobic, strengthening, neuromuscular exercises, and education to support lifestyle modification and home exercise adherence. Outcomes were assessed at baseline, program completion, and longitudinally thereafter; analyses focused on follow-up up to 2 years due to attrition. Primary outcomes were pain (Numeric Pain Rating Scale, NPRS), perceived change (Numeric Global Rating of Change, NGRCS), and knee function (Knee Injury and Osteoarthritis Outcome Score, KOOS).

**Results:**

Participants were stratified by baseline Knee Society Score (KSS): Poor (≤ 66), Fair (67–76), Good (77–89), and Excellent (90–100). Significant improvements were observed across most outcomes in all subgroups. Total KSS and KSS Function scores declined over time, consistent with progression in an end-stage cohort. Benefits were sustained up to 1.5 years in the Poor subgroup, 1 year in the Fair subgroup, and less than 1 year in the Good and Excellent subgroups.

**Conclusions:**

A structured six-session programme was associated with sustained symptom relief in patients awaiting surgery, particularly among those with poorer baseline function. Severity-stratified progression strategies warrant further evaluation in controlled studies.

**Table:** 

**Key Points** • *A scalable six-session physiotherapy-led programme improved pain and functional outcomes in patients with end-stage knee OA awaiting TKR.* • *Benefits were sustained for up to 18 months, particularly among patients with poorer baseline knee function.* • *Baseline severity modified durability of response, suggesting a need for severity-stratified progression and maintenance strategies.*

**Supplementary Information:**

The online version contains supplementary material available at 10.1007/s10067-026-08235-3.

## Background

Knee osteoarthritis (OA), a common degenerative joint disease characterized by ongoing loss of articular cartilage and hyperplasia of bone, affects an estimated 528 million people worldwide as of 2019 [1, 2]. The prevalence of OA increases with age, being as high as 40% in those over age 70 years. OA is more prevalent in females than in males [3]. Recent studies revealed a rise in OA amongst working age groups defined as individuals aged 15 to 64 years, driven largely by lifestyle factors such as obesity and physical inactivity, alongside aging population dynamics [4, 5]. Increased OA incidence has contributed to longer waiting times for total knee replacement (TKR) surgery in Hong Kong public hospitals, with reported waiting periods ranging from 36 to 110 months in non-urgent cases [6]. Such delays present a significant challenge, as poor preoperative health status is associated with the worse postoperative outcomes following TKR [7]. This underscores the necessity for innovative strategies to reduce TKR wait times. Symptoms become more debilitating over time, with common symptoms including knee pain that worsens with activity as well as varus and valgus deformities, joint instability, and pain following prolonged sitting [3, 5, 8].

First-line treatment of knee OA based on international guidelines emphasizes lifestyle management – namely exercise therapy to promote muscle hypertrophy, muscle strengthening, and weight management [8–12]. These interventions aim to reduce joint instability and enhance patients’ tolerance to activity increases [8–12]. Pharmacological treatments, including non-steroidal anti-inflammatory drugs (NSAIDs), are commonly used to address pain but present with side effects, including renal failure and gastrointestinal bleeding [13]. Emerging studies reveal the combination of exercise therapy and whole body vibration with the potential to delay articular cartilage degeneration and consequently improve functional outcomes, which include increased muscle strength, reduced pain, increased functional performance, and increased quality of life, even for end-stage knee OA patients [1, 8, 13]. Structured patient education and neuromuscular exercise programs further support long-term improvements in physical function [14].

Exercise-based interventions also play a critical role in delaying surgical intervention. A longitudinal cohort study by Lawford et al. demonstrated that patients who underwent a three-month education and exercise intervention program for knee and hip OA had a 20% lower probability of undergoing TKR within five years [15]. Given the complications and recognized risks associated with surgery, non-surgical management holds particular importance for patients awaiting TKR [16]. Hawker et al. reported that 13% to 21% of Canadians who received TKR were unwilling to undergo it beforehand, and only 78.1% of 1053 patients achieved a good outcome defined as improvement in symptoms and patient satisfaction [17]. Furthermore, recent evidence highlights a clinical gap in tailored education and preoperative prehabilitation, with structured interventions shown to improve patients’ physical and psychological readiness for surgery [18, 19].

The Structured Non-Operative Treatment Program (SNTP) is therefore proposed as a comprehensive lifestyle management intervention aimed at improving function and reducing symptoms in patients with end-stage knee OA. To our knowledge, no prior study has reported the long-term (≥ 18-month) outcomes of a physiotherapist-led, supervised non-operative program in a large, real-world cohort of end-stage knee OA patients on an extended surgical waitlist (median wait > 36 months). This study hypothesizes that SNTP was associated with symptom improvement and enhanced functional outcomes in this vulnerable patient population.

## Materials and methods

### Study design

This retrospective cohort study included end-stage knee OA patients potentially eligible for TKR in Hong Kong public hospitals from October 2021 to March 2024. 2243 eligible patients entered our SNTP, and clinical data were extracted from a prospectively maintained institutional database.

The SNTP consisted of six supervised exercise and education sessions delivered over a one-year intervention period. All sessions contained aerobic training, resistance exercise, neuromuscular training, and health education training for promoting lifestyle change and home exercise adherence. Outcome measures were assessed before the initial session and at the final session at the end of the one-year intervention period. Longitudinal assessments were conducted one-year post-intervention, subsequently followed by six-month intervals to assess functional and symptomatic outcomes.

## Ethics approval

The study was conducted in accordance with the Declaration of Helsinki and approved by the Joint Chinese University of Hong Kong – New Territories East Cluster Clinical Research Ethics Committee and Prince of Wales Hospital (CRE-2025.619).

## Inclusion and exclusion criteria

Participants recruited were clinically diagnosed with end-stage knee OA according to the European Alliance of Associations for Rheumatology (EULAR) criteria, requiring radiographic evidence of joint damage, significant functional limitations, and severe knee pain [20]. Knee pain was required to be non-inflammatory and not be attributed to other causes such as malignancy. Patients were excluded if they had undergone bilateral total knee replacement, had problems that contraindicated exercise therapy (unstable cardiovascular or respiratory disease), terminal illness, chronic immobility, recent acute myocardial infarction (less than six weeks before the start of the program), or if they refused to participate. Patients were screened using their medical records, physical examination, and standardized questionnaire(s).

## Exercise stratification and prescription

Prior to beginning the program, participants were categorized as either regular exerciser (defined as engagement in a minimum of 30 min of moderate exercise at least 3 days per week for at least the last six months per American College of Sports Medicine (ACSM) Guidelines for Exercise Testing and Prescription, 10th ed (2021) [21]) or assigned to one of six exercise intensity groups based on medical history and recent physical activity, in accordance with the ACSM guidelines (Table S1). Additionally, all participants were thoroughly and independently screened for signs, symptoms, and medical history indicative of cardiovascular, metabolic, and renal disease by clinical records. Cardiovascular disease included such conditions as cardiac surgery (e.g., coronary artery bypass graft, valve surgery), pacemaker or implantable cardiac defibrillator, myocardial infarction, heart failure, stroke, peripheral vascular disease, and coronary artery disease. The type of metabolic disease was categorized as thyroid disease, metabolic syndrome, diabetes mellitus, or endocrine disease that affects metabolism. Type of renal disease included stage 1–5 chronic kidney disease, end-stage renal disease, nephrotic syndrome, and dialysis dependency. Signs and symptoms indicative of cardiovascular, metabolic, or renal disease included angina, palpitations, tachycardia, heart murmur, dyspnoea at rest, dizziness, ankle edema, intermittent claudication, orthopnea, and paroxysmal nocturnal dyspnea. All participants were medically cleared prior to commencing the exercise program.

## Exercise intervention protocol

The exercise intervention consisted of six supervised group (10–12 patients per group) sessions over a year; each session started with 20 min of education, then ~ 30 min of strengthening, aerobic, and neuromuscular exercises, and ~ 30 min reviewing any home exercise program. Strengthening exercises targeted gluteus maximus, quadriceps, hamstrings, hip flexors, hip abductors and calf muscles using body weight, resistance bands, and free weights at moderate intensity (2–3 sets of 8–12 reps), progressing as tolerated and safe. Patients without contraindications also completed 4 sessions of hydrotherapy (30 min of strengthening plus 15 min of aquatic Tai Chi) and, if appropriate, 10 min of WBV with mini squats. Home exercise was prescribed using instructional videos and handouts. Full exercise parameters and educational dosage in Supplementary Table S3.

### Outcome measures

Primary outcomes were patient-reported pain using the Numeric Pain Rating Scale (NPRS), perceived change using Numerical Global Rating of Change Scale (NGRCS), and self-reported function using the Knee Injury and Osteoarthritis Outcome Score (KOOS). KOOS subscale scores range from 0 to 100 – higher scores indicate fewer symptoms and better knee function (Supplementary Table S4). NPRS scores range from 0 to 10, and NGRCS scores range from − 10 to + 10. Secondary outcomes included performance-based functional tests: 30-Second Sit-to-Stand, Timed Up and Go, and Functional Reach Test. Physiotherapists also assessed the Knee Society Score (KSS), focusing on knee stability, alignment, and range of motion as well as the KSS Function Subscale evaluating walking ability, stair climbing, and use of walking aids (descriptions in Supplementary Table S5).

### Statistical analysis

Descriptive statistics were conducted for age, sex, and body mass index (BMI). Paired t-tests compared outcomes before and after SNTP and follow-ups. Subjects were assigned to one of four groups based on KSS at baseline values: Poor (≤ 66), Fair (67–76), Good (77–89), and Excellent (90–100). Levene’s test was used to assess whether or not the variances were equal. One-way ANOVA with Tukey post-hoc testing was used to evaluate differences between subgroups of continuous variables that possessed equal variances. When the variances were deemed unequal, then Welch ANOVA with Games–Howell post-hoc testing was used. A Chi-square test compared sex distribution among sub-groups. Statistical analyses were conducted using IBM SPSS Statistics (Version 30.0), with significance set at p < 0.05.

## Results

**Fig. 1:**
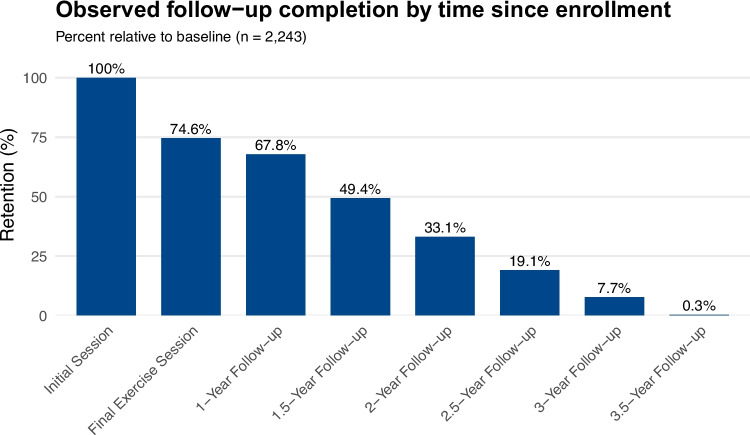
Observed follow-up completion by time since enrollment

Of 2,243 participants, 71.2% (1,597) were female, mean age at entry was 68.4 ± 7.1 years, and the mean BMI was 26.1 ± 4.2 kg/m2 (Table S6). Outcome measures were collected at baseline and at the end of the 6 weeks intervention, and again at 1-year post-intervention and every six months thereafter. As the number of participants contributing data decreased at later follow-up timepoints due to staggered enrollment (Fig. 1), extended analyses were restricted to a maximum of 2 years.

Following SNTP, significant improvements were observed (Table 1) for the NPRS, subscale scores for KOOS, and functional performance tasks. Improvements in pain intensity (NPRS) and lower-limb strength (30-Second Chair Stand) were sustained through the 2-year follow-up. From the follow-ups within 1–1.5 years, several KOOS subscales demonstrated significant improvements, though attenuation was observed thereafter. In contrast, KSS and KSS Function scores declined from baseline at subsequent follow-up time points.

**Table 1:** Longitudinal changes in outcome measures at key follow-up points

Outcome Measure	Final Session (n = 1674) Δ Mean,	p-value	1-Year (n = 1521) Δ Mean	p-value	1.5-Years (n = 1109) Δ Mean	p-value	2-Years (n = 743) Δ Mean	p-value
NPRS	−0.9	< 0.001	−0.6	< 0.001	−0.5	< 0.001	−0.3	< 0.001
KOOS-KP	2.9	< 0.001	2.4	< 0.001	1.7	< 0.001	−0.6	0.167
KOOS-KQ	1.8	< 0.001	1.8	< 0.001	−1.8	0.002	−1.1	0.074
KOOS-Symptoms	1.4	< 0.001	1.7	< 0.001	1.7	0.001	−0.3	0.355
KOOS-ADL	2.4	< 0.001	0.4	0.184	−1.2	0.014	−3.3	< 0.001
KOOS-Sport	5.8	< 0.001	2.2	< 0.001	0.7	0.143	−2.2	0.004
30CST	0.7	< 0.001	0.5	< 0.001	0.5	< 0.001	0.4	0.010
TUG	−1.1	< 0.001	0.0	0.442	0.3	0.068	0.5	0.037
Functional Reach	2.1	< 0.001	0.9	< 0.001	0.1	0.395	0.1	0.371
KSS	−10.0	< 0.001	−16.1	< 0.001	−28.2	0.001	−32.2	< 0.001
KSS-Function	−11.5	< 0.001	−18.3	< 0.001	−30.3	0.001	−39.2	< 0.001

^a^Abbreviations: NPRS, Numeric Pain Rating Scale; KOOS, Knee Injury and Osteoarthritis Outcome Score; KP, Knee Pain; KQ, Knee Quality of life; ADL, Activities of Daily Living; CST, Chair Stand Test; TUG, Timed Up and Go; KSS, Knee Society Score

^b^Δ Mean represents the mean of individual paired differences (follow-up minus baseline). Negative values for NPRS and TUG indicate improvement; negative values for KSS and KSS-Function indicate worsening knee status. Positive values for 30CST and Functional Reach indicate improvement

^c^Changes from baseline were analyzed using paired t tests at each follow-up time point. Analyses were based on participants with available paired baseline and follow-up data for the respective outcome at each time point. All p-values are two-sided

### Baseline Characteristics of each subgroup

Based on the KSS scores, 60.7% of participants were rated Poor, 20.6% Fair, 10.7% Good, and 8.1% Excellent, with significant variance differences across each of the four categories at baseline for BMI, NPRS, KOOS-KP, KOOS-Symptoms, KOOS-ADL, KOOS-Sports and Recreational, 30-s chair stand test, Timed Up-and-Go test, and across KSS (p < 0.05). Sex distribution did not significantly differ among subgroups. Due to baseline functional disparities, subgroup analyses were separately conducted to better evaluate the SNTP effects. The poor function group has significant higher BMI and the and older age (Table S6).

### Longitudinal changes in outcome measures at key follow-up points

NGRCS scores differed significantly among subgroups beginning at the 1‑year follow‑up and continuing through subsequent follow‑up periods (Table 2). Across all subgroups, mean NGRCS scores declined following completion of the 6‑session program. However, at each follow‑up time point, participants classified as Good and Excellent at baseline consistently demonstrated higher mean NGRCS scores compared with those in the Poor and Fair subgroups, indicating more favorable perceived global change among higher-functioning participants.

**Table 2:** Comparison of NGRCS at each follow-up session among subgroups

Follow-up Session	Poor (Mean ± SD)	Fair (Mean ± SD)	Good (Mean ± SD)	Excellent (Mean ± SD)	P-value
Final Exercise Session	2.2 ± 2.3	2.4 ± 2.5	2.6 ± 2.6	2.6 ± 2.6	0.129
1-Year Follow-up	0.1 ± 3.2	0.3 ± 3.2	1.1 ± 3.1	1.4 ± 3.2	< 0.001
1.5-Year Follow-up	0.4 ± 3.1	1.0 ± 3.2	1.5 ± 2.9	1.4 ± 3.5	< 0.001
2-Year Follow-up	−0.4 ± 3.2	−0.08 ± 3.3	0.5 ± 3.4	0.5 ± 3.2	0.036

Between-group comparisons were performed. All *p*-values are two-sided

When examining overall trends (Table 1**, **S7), immediate post‑intervention improvements were observed across most outcome measures. Pain (NPRS) decreased from 5.60 (2.08) at baseline to 4.68 (2.09) at the final session. Improvements were also observed in KOOS subscales, including Knee Pain, Symptoms, ADL, and Sports/Recreation. Functional performance measures improved modestly, with increases in 30‑Second Chair Stand repetitions and reductions in Timed Up and Go time. These gains were generally maintained through 1‑year follow‑up, with slight attenuation by 1.5 to 2 years. In contrast, KSS demonstrated a progressive decline over time despite short‑term improvements in patient‑reported and functional outcomes.

### Poor subgroup

Among the 1362 participants with a KSS score of ≤ 66, the 6 group sessions consisting of exercise and knee education resulted in statistically significant improvements in pain, KOOS subscales, lower limb function (30-Second Chair Stand and Timed Up and Go), and balance (Functional Reach), with the improvement in NPRS reaching minimal clinically important difference (MCID). In contrast, both KSS and the KSS Function subscale declined over time. Intervention benefits were sustained for up to 1.5 years (see Supplementary Table S8).

### Fair subgroup

Among the 461 participants with baseline KSS scores between 67 and 76, statistically significant improvements were observed immediately after the 6 group sessions; however, none reached the MCID. Similar to the poor subgroup, significant declines were observed in the KSS and KSS Function Subscale scores. Treatment benefits were not sustained 1 year after the initial assessment (see Supplementary Table S8**)**.

### Good subgroup

Among the 239 participants with baseline KSS between 77 and 89, statistically significant improvements were observed in approximately half of the outcome measures, accompanied by declines in KSS and KSS Function subscale scores. SNTP benefits are substantially diminished by 1–1.5 years (see Supplementary Table S8).

### Excellent subgroup

Among the 181 participants with baseline KSS ≥ 90, a similar pattern was observed, with statistically significant improvements in approximately half of the outcomes and declines in both KSS scores. SNTP improvements following 1 year were largely absent (see Supplementary Table S8).

## Discussion

In this large real-world cohort of end-stage knee OA patients awaiting TKR, participation in a six-session SNTP delivered over one year was associated with improvements in pain and multiple patient-reported and performance-based outcomes, with selected benefits persisting for up to 18 months and, for some measures, up to two years among participants who attended follow-up. These findings extend existing exercise evidence to an end-stage surgical-waitlist population using a scalable program model. Notably, durability differed by baseline knee status, supporting the need for context-specific progression and maintenance strategies. These improvements align with previous research demonstrating that structured exercise leads to symptomatic benefits in knee OA. Significantly, those in a group that received in-person supervised physiotherapy were almost five times more likely to adhere than those using an exercise app (OR 4.2, 95% CI 1.5–12.4) (Martinsen et al. 2025), suggesting an important role for physiotherapist engagement to mitigate gaps in rehabilitation adherence [22]. At the same time, others have shown high adherence to self-directed exercise programmes and structured digital education [23]. Together, these findings suggest that structured guidance, accountability, and behavioral reinforcement play key roles in sustaining engagement. Our SNTP program appears to reflect these principles by combining supervised physiotherapist-led sessions with a structured exercise program, which may support sustained engagement during the waiting period for surgery.

Patients with a poor baseline knee function (KSS ≤ 66) achieved the most sustained benefits – specifically up to 1.5 years. Whereas patients with fair, good, and excellent baseline function demonstrated more gradual and less pronounced improvements, and hence require more progressive overload and individualized progression in order to see more sustained benefits. This reinforces the study by Martinsen et al. (2025), where adherence alone did not fully predict outcomes [22]. Taken together, these findings suggest that baseline functional status may modify not only the magnitude but also the durability of response, implying that a uniform program may preferentially benefit those with lower initial function while higher baseline groups may require greater progression and/or maintenance contacts. Several non-mutually-exclusive mechanisms may explain why participants with poor baseline KSS achieved the most sustained benefits. First, a floor effect: those with low baseline scores had greater 'room to improve' on every outcome. Second, a physiological dose–response effect: lower baseline muscle strength, lower habitual physical activity, and greater functional deficits are known to produce larger relative training responses for a given exercise stimulus, meaning the same six-session program likely delivered a proportionally stronger neuromuscular signal in the poor group.

A notable pattern in our data was sustained improvements in pain and selected performance-based outcomes alongside a progressive decline in clinician-assessed KSS and KSS Function scores over time. This should be interpreted in the clinical context of an end-stage cohort already on a trajectory toward arthroplasty. KSS is a composite clinician-rated measure influenced by pain, alignment, stability, range of motion, and functional domains such as walking and stair climbing; therefore, declining KSS may reflect broader changes in overall knee status and functional capacity that can continue to worsen in severe disease despite concurrent symptom relief and improvements in performance tests. In this setting, SNTP may offer clinically meaningful symptom relief and functional preservation during a period when deterioration in global knee status is expected. This is consistent with observations from Kong et al. (2025), where structured exercise and patient education were also implemented in the modified GLA:D® programs [24].

Given the long waiting duration for TKR in Hong Kong, SNTP offers a valuable strategy to alleviate symptoms and enhance the function of end-stage OA knee patients during this period. However, modifications to the program design may be necessary to sustain the benefits of the program for over 1.5 years. From a health-system perspective, a scalable, group-based program that improves pain and function during prolonged TKR waiting times may mitigate deconditioning and functional decline and may improve patients’ readiness for eventual surgery. Moreover, the SNTP is not a substitute for TKR in end-stage knee OA, and the progressive decline in clinician-rated KSS observed across all subgroups confirms that the underlying joint disease continues to advance despite symptomatic improvement. Rather, our findings support SNTP as a bridging intervention that may relieve pain, preserve function, and mitigate deconditioning during the prolonged interval before surgery becomes available.

The SNTP intervention was implemented in a real-world setting that is scalable in public healthcare systems with significantly long TKR wait times. The non-invasive nature of the program, incorporating neuromuscular training, resistance training, and aerobic exercise, makes SNTP cost-efficient and feasible to be implemented across diverse healthcare settings. Furthermore, a comprehensive evaluation of pain, function, and physical performance was achieved through the evaluation of both subjective (e.g., NPRS) and objective (e.g., KSS) outcome measures. The integration of supervised physiotherapist-led sessions with structured home self-directed exercises is another strength supported by Souto et al. (2025), as this blended approach promotes patient autonomy under continuous professional guidance, achieving greater adherence than self-directed exercises alone [23]. In addition, stratification by baseline KSS enabled clinically interpretable comparisons of benefit durability across severity strata, which may help inform program tailoring in future implementation.

This study has several limitations. First, the absence of a control group limits causal inference, and observed improvements cannot be directly attributed to the SNTP intervention. Second, unmeasured confounding factors, including independent exercise participation outside the program, natural symptom fluctuation, and regression to the mean, cannot be excluded. Third, subgroup sample sizes were unequal, with a substantially larger proportion of participants in the poor baseline KSS category compared with the fair, good, and excellent groups, which may have influenced statistical precision and comparability across strata. Fourth, imaging-based structural endpoints were not collected. Therefore, the observed decline in clinician-assessed KSS over time should be interpreted as a change in overall clinical knee status in an end-stage cohort awaiting arthroplasty, rather than direct evidence of structural disease progression. Finally, substantial attrition reduced the availability of follow-up data beyond two years and may have introduced selection bias, as participants returning for follow-up may not have been fully representative of the original cohort. Despite these limitations, the large sample size and consistent longitudinal trends strengthen the overall interpretability of the findings.

Further studies investigating whether tailored exercise programs that consider patients’ baseline function and motivation profiles can improve adherence (ideally along with clinical outcomes) would be worthwhile. Automated embedding of behavioral support features in the self-guided element of the program, such as tracking progress, setting goals, or remote monitoring via fitness device or app, may further improve compliance and long-term engagement. Additionally, studies are needed to determine if the effects of structured exercise programs can be sustained beyond 1.5 years and work towards understanding the potential of blended approaches for OA exercise programs in which patients have some traditionally delivered follow-up care and some digitally delivered guidance. Further, to better discern causes of observed changes, pragmatic study designs and the measurement of adherence, co-interventions, and objective activity levels could provide clarity on which program components induce durable outcomes and for whom. Future program refinements could include severity-stratified dosing, standardized progression algorithms, and booster sessions to extend benefits beyond 12–18 months, particularly in higher baseline function groups.

Overall, this study demonstrates that a structured, six-session non-operative exercise program combined with health education can provide sustained symptom relief and functional benefit in patients with end-stage knee OA, with the greatest durability observed among those with poorer baseline functional status. These findings suggest that maintenance and progression strategies may benefit from severity-based stratification, whereby follow-up intensity and exercise progression are tailored according to baseline functional capacity. While further controlled studies are required to confirm long-term efficacy and causal effects, structured exercise programs may serve as a pragmatic approach to mitigate symptom burden during prolonged waiting periods for TKR.

## Supplementary Information

Below is the link to the electronic supplementary material.

ESM 1 (DOCX 3.33 MB)

## Data Availability

The data that support the findings of this study are available from the corresponding author upon reasonable request.
