# Supplementary material for: Six-session non-operative exercise program yields sustained benefits for up to 18 months in end-stage knee osteoarthritis: a retrospective cohort study
Source: Clin Rheumatol. 2026 Jun 27;45(8):5055–62. doi: 10.1007/s10067-026-08235-3 (PMC13486104; doi:10.1007/s10067-026-08235-3)
Supplement: Supplementary file 1 [file 10067_2026_8235_MOESM1_ESM.docx]

**Supplementary Files**

**Title:** Six-session non-operative exercise program yields sustained benefits for up to 18 months in end-stage knee osteoarthritis

**Authors:** Wing Yip Lee^1^, Linda Man Kuen Li^1^, Naomi Cheuk Ying Chen^2^, Chloe Sze Man Leung^1^, Alan Yin Chung Tsui^1^, Xueyou Zhang^2^, Mingde Cao^2^, Patrick Shu-Hang Yung^2,3^, Michael Tim-Yun Ong^2,3^

^1^Department of Physiotherapy, Prince of Wales Hospital, Hong Kong SAR, China.

^2^Department of Orthopaedics and Traumatology, Chinese University of Hong Kong, Hong Kong SAR, China.

^3^CUHK Medical Centre, Department of Orthopaedics & Traumatology, Faculty of Medicine, The Chinese University of Hong Kong.

This file contains Supplementary Tables S1-S8 referenced in the manuscript.

**Table S1:** Exercise stratification based on medical status and exercise habits according to ACSM guidelines, 10th edition (2018)

| Category | Exercise Habit | Medical Status | Medical Clearance | Recommended Exercise | Progression |
| --- | --- | --- | --- | --- | --- |
| 1 | Does not exercise regularly | No CV, metabolic, or renal disease AND no S/S in last 12 months | Not necessary | Light to moderate intensity | May gradually progress to vigorous |
| 2 | Does not exercise regularly | Known CV, metabolic, or renal disease AND no S/S in last 12 months | Recommended | Light to moderate intensity | May gradually progress as tolerated |
| 3 | Does not exercise regularly | S/S suggestive of CV, metabolic, or renal disease (any status) | Recommended | Light to moderate intensity | May gradually progress as tolerated |
| 4 | Exercises regularly | No CV, metabolic, or renal disease AND no S/S in last 12 months | Not necessary | Moderate or vigorous intensity | May gradually progress |
| 5 | Exercises regularly | Known CV, metabolic, or renal disease AND no S/S in last 12 months | Not necessary for moderate; clearance needed for vigorous if no change in S/S | Continue with moderate; may engage in vigorous after clearance | May gradually progress as tolerated |
| 6 | Exercises regularly | Any S/S suggestive of CV, metabolic, or renal disease (any status) | Discontinue exercise and seek clearance | Resume after clearance | May gradually progress as tolerated |

**Table S2:** Classification of exercise intensity levels based on heart rate reserve (HRR), rate of perceived exertion (RPE), and metabolic equivalent of task (MET) according to ACSM guidelines

| Exercise Intensity | HRR (%) | RPE (Borg 6–20) | METs |
| --- | --- | --- | --- |
| Light | 30–39 | 9–11 | 2.0-2.9 |
| Moderate | 40–59 | 12–13 | 3.0-5.9 |
| Vigorous | ≥ 60 | ≥ 14 | ≥ 6.0 |

**Table S3:** Educational topics covered in the SNTP

| Lesson | Content |
| --- | --- |
| 1 | Knee anatomy, knee OA disease, symptoms and risk factors, the role of exercise and aims of OA management and introduction to home exercise program. |
| 2 | Benefits of exercise, strategies to incorporate exercise to into daily routine, map of local exercise facilities available in community |
| 3 | Diet, body mass index and sarcopenia, balanced nutrition and use of diet and exercise diaries to monitor progress |
| 4 | Acute and chronic pain physiology; exacerbation factors and self-management techniques including relaxation skills |
| 5 | Introduction to the TKR procedure, pre/post operative process, and importance of ongoing exercise. Video on what to expect with TKR shown |

**Table S4:** KOOS subscales and their descriptions

| KOOS Outcome | Explanation |
| --- | --- |
| KOOS - Pain | Intensity and frequency of knee pain |
| KOOS - Physical Function | Limitations in performing physical tasks |
| KOOS - Quality of Life (QoL) | Patient’s perception of their knee condition and its impact on their mental, social, and emotional well-being |
| KOOS - Symptoms | Symptoms include swelling and stiffness |
| KOOS - Sports/Recreation | Difficulty of performing sport and recreation related activities |
| KOOS - Activities of Daily Living (ADL) | Difficulty of common daily tasks such as standing, walking, and climbing the stairs |

**Table S5**: Functional performance-based tests and their descriptions

| Performance-Based Test | Explanation |
| --- | --- |
| 30-second Sit-to-Stand (30s-STS) | Counts the number of times the patient can rise from a seated position in 30 seconds[1]. |
| Timed Up and Go (TUG) | Measures the time a patient can stand from a seated position, walk three meters, turn, and sit back down[2]. |
| Functional Reach Test | Measures the distance the patient can reach forward without losing balance[3]. |
| Knee Society Score (KSS) | Clinician-administered score which rates the range of motion, knee stability, and knee alignment. |
| KSS Function subscale | Clinician-administered score which rates the patient's walking distance, climbing stairs and use of walking aids. |

**Table S6:** Baseline comparison of outcomes of each subgroup

|  | Poor | Fair | Good | Excellent | p-value |
| --- | --- | --- | --- | --- | --- |
| Age (years) | 68.8 ± 7.0 | 68.1 ± 7.2 | 67.9 ± 6.9 | 67.5 ± 7.7 | 0.03 |
| Number of males (n, %) | 370 (27.1%) | 137 (29.7%) | 83 (34.7%) | 57 (31.5%) | 0.291 |
| BMI (kg/m²) | 26.6 ± 4.4 | 25.5 ± 4.0 | 25.7 ± 4.0 | 24.7 ± 3.3 | <0.001 |

**Table S7:** Longitudinal changes in outcome measures at key follow-up points for participants

| **Outcome Measure** | **Baseline Mean (SD)** | **Final Session, Mean (SD)** | **1-Year**  **Mean (SD)** | **1.5-Year**  **Mean (SD)** | **2-Year**  **Mean (SD)** |
| --- | --- | --- | --- | --- | --- |
| NPRS (pain) | 5.60 (2.08) | 4.68 (2.09) | 4.94 (2.13) | 4.94 (2.13) | 5.12 (2.19) |
| KOOS - Knee Pain (KOOS-KP) | 53.4 (18.9) | 56.1 (18.7) | 56.4 (20.1) | 56.3 (20.0) | 54.1 (19.9) |
| KOOS-Knee Quality (KOOS-KQ) | 37.7 (20.8) | 39.2 (20.2) | 39.6 (20.9) | 39.9 (20.6) | 37.3 (20.5) |
| KOOS-Symptoms | 53.2 (21.9) | 54.3 (21.6) | 55.6 (22.3) | 55.7 (22.0) | 53.0 (22.2) |
| KOOS – Activities of Daily Living (KOOS-ADL) | 64.1 (21.0) | 66.5 (19.7) | 65.2 (21.1) | 64.2 (21.2) | 61.9 (21.5) |
| KOOS-Sports | 33.8 (24.4) | 39.5 (25.0) | 36.8 (25.4) | 35.8 (25.0) | 32.5 (23.8) |
| 30-Second Chair Stand (30CST) | 7.62 (3.75) | 8.34 (3.77) | 8.22 (4.39) | 8.34 (3.86) | 8.47 (5.07) |
| Timed Up and Go (TUG) | 14.5 (7.75) | 13.2 (6.30) | 13.9 (6.91) | 14.3 (8.92) | 14.5 (8.59) |
| Functional Reach | 23.1 (7.64) | 25.1 (7.95) | 24.1 (7.32) | 23.3 (6.98) | 23.4 (7.32) |
| Knee Society Score (KSS) | 55.2 (24.6) | 46.9 (33.8) | 44.2 (33.8) | 32.0 (34.7) | 27.9 (33.1) |

**Table S8:** Longitudinal changes in outcome measures at key follow-up points for participants with poor (≤66) , fair (67 to 76) , good (77 to 89), and excellent (≥90) KSS baseline

| **Outcome Measure** | **Subgroup** | **Baseline Mean (SD)** | **Δ Final Session, p-value** | **Δ 1-Year, p-value** | **Δ 1.5-Years, p-value** | **Δ 2-Years, p-value** |
| --- | --- | --- | --- | --- | --- | --- |
| NPRS (pain) | Poor | 6.6±1.7 | -1.2, <0.001 | -1.0 , <0.001 | -1.1 , <0.001 | -0.9 , <0.001 |
|  | Fair | 4.9±1.5 | -0.6, <0.001* | -0.4, 0.00* | -0.1, 0.272 | -0.0, 0.457 |
|  | Good | 3.3±1.3 | -0.1, 0.134 | 0.3, 0.007* | 0.3, 0.044* | 0.8, 0.001* |
|  | Excellent | 3.0±1.3 | -0.3, 0.003* | 0.5, 0.003* | 0.9, <0.001* | 0.9, 0.001* |
| KOOS - Knee Pain (KOOS-KP) | Poor | 47.1±18.0 | 3.4, <0.001* | 3.1, <0.001* | 2.7 , <0.001* | 1.0, 0.237 |
|  | Fair | 58.2±15.8 | 2.5, 0.001* | 2.5, 0.002* | 1.5 , 0.082 | -1.0 , 0.218 |
|  | Good | 66.7±15.5 | 1.3, 0.087 | 0.3, 0.418 | -1.7 , 0.132 | -5.8 , 0.002* |
|  | Excellent | 70.6±13.2 | 2.1, 0.019* | -0.6 , 0.319 | -0.3 , 0.423 | -2.9 , 0.054 |
| KOOS-Knee Quality (KOOS-KQ) | Poor | 32.7±19.4 | 1.8, <0.001* | 2.3 , <0.001* | 2.8 , <0.001* | -0.7 , 0.474 |
|  | Fair | 41.3±19.5 | 2.0, 0.019* | 2.2 , 0.026* | 0.3 , 0.411 | -0.6 , 0.355 |
|  | Good | 48.3±21.3 | 0.4, 0.384 | 0.6 , 0.333 | 1.0 , 0.298 | -0.9 , 0.36 |
|  | Excellent | 52.6±20.2 | 2.9, 0.04* | -0.9 , 0.338 | -1.3 , 0.298 | -4.9 , 0.032* |
| KOOS-Symptoms | Poor | 46.6±20.7 | 2.0, <0.001* | 2.4 , <0.001* | 3.3 , <0.001* | 1.6 , 0.119 |
|  | Fair | 59.0±20.3 | 0.8, 0.184 | 1.9 , 0.034* | 0.5 , 0.354 | -1.6 , 0.135 |
|  | Good | 65.8±17.8 | 0.1, 0.472 | 0.4 , 0.364 | -1.8 , 0.121 | -4.3 , 0.027* |
|  | Excellent | 71.3±17.1 | -0.2, 0.454 | -1.8 , 0.097 | -1.5 , 0.186 | -2.7 , 0.102 |
| KOOS – Activities of Daily Living (KOOS-ADL) | Poor | 57.8±21.1 | 3.1, <0.01* | 1.2 , 0.019* | -0.1 , 0.46 | -1.6 , 0.066 |
|  | Fair | 69.4±17.7 | 2.0, 0.004* | 0.0 , 0.497 | -2.2 , 0.014* | -3.6 , 0.001* |
|  | Good | 76.7±15.3 | -0.2, 0.411 | -1.7 , 0.056 | -4.0 , 0.001* | -8.4 , <0.001* |
|  | Excellent | 80.6±13.2 | 1.3, 0.105 | -2.0 , 0.102 | -2.4 , 0.089 | -6.2 , 0.005* |
| KOOS-Sports | Poor | 27.2±21.4 | 5.9, <0.001* | 2.6 , <0.001* | 0.9 , 0.159 | -2.3 , 0.024* |
|  | Fair | 38.3±23.5 | 6.6 , <0.001* | 2.4  , 0.031* | 1.1 , 0.214 | 0.6 , 0.357 |
|  | Good | 48.2±25.5 | 3.3 , 0.019* | 1.1 , 0.262 | -0.7 , 0.361 | -4.3 , 0.048 |
|  | Excellent | 53.2±25.1 | 5.8 , 0.001* | 0.6 , 0.382 | 0.7 , 0.402 | -5.3 , 0.049* |
| 30-Second Chair Stand (30CST) | Poor | 6.9±3.8 | 0.8 , <0.001* | 0.7 , <0.001* | 0.6 , <0.001* | 0.6 , 0.034* |
|  | Fair | 8.2±3.4 | 0.4 , 0.002* | 0.4 , 0.017* | 0.3 , 0.086 | 0.4 , 0.066 |
|  | Good | 9.1±3.6 | 0.4 , 0.02* | 0.0 , 0.441 | 0.3 , 0.161 | 0.2 , 0.325 |
|  | Excellent | 9.6±3.3 | 0.5 , 0.009* | 0.0 , 0.453 | 0.3 , 0.198 | -0.3 , 0.222 |
| Timed Up and Go (TUG) | Poor | 15.8±8.8 | -1.2 , <0.001* | -0.1 , 0.306 | 0.2 , 0.317 | 0.2 , 0.628 |
|  | Fair | 13.4±6.0 | -1.0 , <0.001* | -0.0 , 0.435 | 0.5 , 0.113 | 0.7 , 0.085 |
|  | Good | 12.0±4.9 | -0.6 , 0.017* | 0.1 , 0.349 | 0.7 , 0.009* | 1.4 , 0.016* |
|  | Excellent | 11.1±3.5 | -0.8 , 0.001* | 0.6 , 0.032* | 0.7 , 0.016* | 0.4 , 0.126 |
| Functional Reach | Poor | 22.2±7.5 | 1.9 , <0.001* | 0.9 , 0.002* | -0.0 , 0.452 | -0.2 , 0.759 |
|  | Fair | 24.0±7.5 | 1.7 , <0.001* | 0.7 , 0.084 | -0.1, 0.462 | 1.0 , 0.086 |
|  | Good | 24.9±8.2 | 3.0 , <0.001* | 2.3 , <0.001* | 0.5 , 0.261 | -0.8 , 0.269 |
|  | Excellent | 26.1±6.7 | 2.7 , <0.001* | -0.4 , 0.3 | 0.6 , 0.247 | 0.5 , 0.313 |
| Knee Society Score (KSS) | Poor | 48.3±13.3 | -4.3 , <0.001* | -10.0 , <0.001* | -20.6 , <0.001* | -25.5 , <0.001* |
|  | Fair | 70.3±2.8 | -16.3 , <0.001* | -21.0 , <0.001* | -35.2 , <0.001* | -35.8 , <0.001* |
|  | Good | 82.9±3.7 | -20.3 , <0.001* | -25.7 , <0.001* | -38.9 , <0.001* | -49.4 , <0.001* |
|  | Excellent | 92.9±2.4 | -30.0 , <0.001* | -35.6 , <0.001* | -52.7 , <0.001* | -49.6 , <0.001* |
| KSS Function Subscale | Poor | 56.5±17.9 | -11.5 , <0.001* | -17.1 , <0.001* | -28.6 , <0.001* | -37.2 , <0.001* |
|  | Fair | 64.4±16.5 | -14.8 , <0.001* | -19.5 , <0.001* | -31.9 , <0.001* | -39.4 , <0.001* |
|  | Good | 70.2±16.7 | -14.5 , <0.001* | -18.4 , <0.001* | -30.1 , <0.001* | -43.6 , <0.001* |
|  | Excellent | 76.2±16.6 | -22.1 , <0.001* | -24.3 , <0.001* | -39.1 , <0.001* | -47.0 , <0.001* |

**Reference**

1.	Jones CJ, Rikli RE, Beam WC. A 30-s chair-stand test as a measure of lower body strength in community-residing older adults. *Res Q Exerc Sport*. 1999;70(2):113–9.

2.	Podsiadlo D, Richardson S. The timed "Up & Go": a test of basic functional mobility for frail elderly persons. *J Am Geriatr Soc*. 1991;39(2):142–8.

3.	Duncan PW, Weiner DK, Chandler J, Studenski S. Functional reach: a new clinical measure of balance. *J Gerontol*. 1990;45(6):M192–7.
